# Adiponectin agonist treatment in diabetic pregnant rats

**DOI:** 10.1530/JOE-20-0617

**Published:** 2021-06-22

**Authors:** Antonio Gázquez, Francisca Rodríguez, María Sánchez-Campillo, Lidia E Martínez-Gascón, Marino B Arnao, Pedro Saura-Garre, María D Albaladejo-Otón, Elvira Larqué

**Affiliations:** 1 Department of Physiology, CEIR Campus Mare Nostrum, University of Murcia, Biomedical Research Institute of Murcia, Murcia, Spain; 2 Department of Clinical Analysis, Biomedical Research Institute of Murcia, Santa Lucia General University Hospital, Murcia, Spain; 3 Department of Plant Biology (Plant Physiology), University of Murcia, Murcia, Spain; 4 Department of Clinical Psychology, University Clinical Hospital Virgen de la Arrixaca, Murcia, Spain

**Keywords:** diabetes, adiponectin, DHA, glucose, pregnancy

## Abstract

Gestational diabetes mellitus (GDM) reduces maternal adiponectin and docosahexaenoic acid (DHA) materno-fetal transfer, which may have negative consequences for the offspring. Our aim was to evaluate the effects of the administration of a novel adiponectin agonist (AdipoRon) to GDM rats on the long-term consequences in glycaemia and fatty acids (FA) profile in the offspring. Pregnant rats were randomized to three groups: GDM rats (GDM, *n* = 8), GDM rats treated with AdipoRon (GDM + ADI, *n* = 9), and control rats (*n* = 10). Diabetes was induced with streptozotocin (50 mg/kg) on day 12 of gestation. GDM+ADI received 50 mg/kg/day AdipoRon from day 14 until delivery. Glycaemia and FA profile were determined in mothers and adult offspring (12 weeks old). AdipoRon tended to reduce fasting glucose in diabetic mothers. Diabetic rats presented the foetus with intrauterine growth restriction and higher adiposity, which tried to be counteracted by AdipoRon. In the adult offspring, both GDM + ADI and control animals showed better glucose recovery after oral glucose overload with respect to GDM. DHA in offspring plasma was significantly reduced in both GDM and GDM + ADI compared to controls (*P* = 0.043). Nevertheless, n-6/*n*-3 polyunsaturated FA (PUFA) ratio improved in plasma of GDM + ADI adult offspring (GDM: 14.83 ± 0.85a%; GDM + ADI: 11.49 ± 0.58b%; control: 10.03 ± 1.22b%, *P* = 0.034). Inflammatory markers and oxidative stress were reduced in the adult offspring of AdipoRon-treated mothers. In conclusion, AdipoRon administration to pregnant diabetic rats improved glycaemia in the mothers and long-term glucose tolerance in the offspring. In addition, it tended to reduce excessive foetal fat accumulation and improved n-6/n-3 PUFA ratio significantly in offspring at the adult state.

## Introduction

Gestational diabetes mellitus (GDM) prevalence is increasing in women due to the obesity epidemic; however, the ideal management of GDM remains controversial (Mack & Tomich 2017). It is estimated that one out of six live births is affected by hyperglycaemia in pregnancy, most of those due to GDM (International Diabetes Federation 2017). GDM is associated with neonatal adverse outcomes such as large for gestational age infants and increased risk of obesity and cardiometabolic disease later in life (Boney *et al.* 2005, Lawlor *et al.* 2010), contributing to the perpetuation of the vicious cycle of obesity, diabetes, and metabolic syndrome. There is also growing evidence of the GDM long-term consequences for mothers, who have a higher risk of developing type 2 diabetes and cardiovascular disease (Bellamy *et al.* 2009, Kramer *et al.* 2019).

Several cohort studies reported decreased adiponectin concentrations in GDM women compared to their peers without diabetes (Fasshauer* et al.* 2014). Adiponectin is an insulin-sensitizing adipokine that promotes FA oxidation (liver and muscle) and lowers the expression of genes involved in hepatic gluconeogenesis via activation of AMP-activated protein kinase (AMPK) and via peroxisome proliferator-activated receptor *α* (PPAR*α*) (Yamauchi *et al.* 2001, Kadowaki & Yamauchi 2005). Low adiponectin levels combined with high insulin resistance in GDM might contribute to enhance GDM-associated macrosomia (Fasshauer* et al.* 2014). Moreover, hypoadiponectinemia in pregnancy is a predictor of postpartum insulin resistance, beta-cell dysfunction, and elevated fasting glycaemia and may play a role in the subsequent development of type 2 diabetes (Retnakaran* et al.* 2010). These observations raised the question about the utilization of adiponectin as a new therapeutic target for insulin resistance in diabetic subjects.

There are two strategies to reverse hypoadiponectinemia, one is to increase adiponectin concentration itself through the injection of adiponectin which presents some methodological problems due to its very high concentration in blood and the high molecular weight of the active form (Pajvani* et al.* 2004). Other option is the use of adiponectin receptors (AdipoR1 and AdipoR2) agonists that mimic adiponectin effects. Adiponectin administration in mice models has shown positive effects on insulin sensitivity (Yamauchi *et al.* 2001, Combs *et al.* 2004) as well as using adiponectin agonists (AdipoRon) in high-fat diet obese mice (Okada-Iwabu* et al.* 2013). However, little is known about the effects of AdipoRon administration during gestation, especially in those pregnancies affected by GDM.

Several studies have shown impaired placental transport of docosahexaenoic acid (22:6 n-3, DHA) by GDM (Herrera & Ortega-Senovilla 2010, Leveille *et al.* 2018), likely as a consequence of alterations in fatty acids (FA) transport proteins related to phospholipids transfer such as the major facilitator superfamily domain-containing 2a (MFSD2a) and FA transport protein 4 (FATP-4) (Prieto-Sanchez *et al.* 2017, Segura *et al.* 2017). Insufficient supply of long-chain polyunsaturated FA (LC-PUFA) to the foetus might compromise adequate growth and later on visual and cognitive development (Innis 2007).

The aim of the present study was to investigate the effects of AdipoRon administration during gestation on glycaemia and lipid profile in both mothers and adult offspring using a rat model of GDM.

## Materials and methods

### Animals and study design

All procedures were approved by the Institutional Animal Care and Use Committee of the University of Murcia (Murcia, Spain) and conformed to the ARRIVE guidelines for animal research (Kilkenny* et al.* 2010). Animals received humane treatment in accordance with the European Union guidelines for the care and use of laboratory animals. Female adult Sprague Dawley rats (12 weeks of age) were supplied by the Animal Laboratory Service of the University of Murcia. Average weight of rats was 225 ± 5 g. Animals were housed individually with access to food and water *ad libitum* in a humidity and temperature-controlled (22 ± 1°C) room on a 12 h light:12 h darkness cycle.

Female rats were mated (1:1), and the first day of gestation was estimated by the presence of a mating plug in the cage. They were randomly assigned to three groups of 15 animals each: diabetic rats (GDM), diabetic rats treated with adiponectin agonist (AdipoRon, Biorbyt, UK) (GDM + ADI), and healthy controls without diabetes (control). Diabetes was induced on day 12 of gestation by a single injection of streptozotocin (STZ) (50 mg/kg i.p.) (Sigma–Aldrich) dissolved in citrate buffer (10 mM, pH 4.5), control animals received vehicle only. Once the diabetes was established, GDM+ADI animals received AdipoRon (50 mg/kg/day by gavage) freshly dissolved in ethanol for days 14–20 of gestation. All diabetic rats (GDM and GDM + ADI) were given exogenous neutral protamine Hagedorn insulin (Novo Nordisk) every morning in order to maintain glycaemia values of mothers between 100 and 400 mg/mL. The units of insulin administered to pregnant rats varied depending on daily glucose values but were similar in both GDM and GDM + ADI groups (GDM: 3.44 ± 0.30 IU/d, GDM + ADI: 3.63 ± 0.33 IU/d, *P* = 0.653).

On day 20 of gestation, ten pregnant rats per group were anesthetized with a mixture (per 100 g) of 5 mg ketamine hydrochloride, 0.25 mg chlorobutanol, and 1 mg xylazine. Maternal blood was extracted by heart puncture in EDTA-coated tubes and centrifugated at 1400 ***g*** for 10 min at 4°C to obtain plasma. Foetal plasma was not possible to collect due to methodological difficulties related to the small size of the foetus at delivery. Placentas, whole foetus, and foetal brain were also collected (four foetus and placentas were pooled per rat), frozen in liquid nitrogen and stored at −80°C until analysis.

Three to four pregnant rats per group were allowed to breed their pups until weaning (21 days). Sex-matched offspring animals were maintained on a standard chow diet until adult state (12 weeks of age) when they were sacrificed, and plasma and brain samples collected (GDM *n* = 16 (8 males/8 females), GDM + ADI *n* = 14 (6 males/8 females), control *n* = 11 (6 males/5 females)). Rats were allocated during 4 days in individual metabolic cages for the collection of urine samples. Oral glucose tolerance test was performed in the offspring at 3 months of age (2 g/kg), blood was collected by a tail puncture at basal, 30, 60, and 90 min postchallenge.

### Biochemical analysis

Plasma biochemical parameters (glucose, total cholesterol, HDL cholesterol, and triglycerides were quantified by an automatic analyser (Roche–Hitachi Modular PyD Autoanalyzer, Mannheim, Germany). Daily monitoring of glucose in diabetic mothers was performed with Ascensia Breeze 2 blood glucose monitoring system (Bayer AG). HbAlc was measured with A1C Now+ Multi-test System (Bayer AG).

### Fatty acids analyses

Total lipids were extracted from 125 μL plasma and tissues (150 mg placenta, 600 mg whole foetus, and 80 mg brain) into chloroform:methanol (2:1 v/v) according to Folch *et al.* method (Folch* et al.* 1957). Previous to the extraction, 0.05 mg pentadecanoic acid was added to the samples as internal standard. FA methyl esters were produced according to Stoffel *et al.* (Stoffel* et al.* 1959) by adding 1 mL of 3 N methanolic HCl (Supelco, Sigma–Aldrich) and heating at 90°C for 1 h. The derivatives were extracted into hexane and stored at −20°C until gas chromatographic analysis.

FA methyl esters were analysed by gas chromatography using a SP-2560 capillary column (100 m × 0.25 mm i.d. × 20 µm) (Supelco, Sigma–Aldrich) in a Hewlett-Packard 6890 gas chromatograph (Agilent Technologies) equipped with a flame ionization detector (Larque* et al.* 2003). The temperature of the detector and the injector was 240°C. The oven temperature was programmed at 175°C for 30 min and increased at 2°C/min to 230°C and held at this temperature for 17 min. Helium was used as the carrier gas at a pressure of 45 psi. Peaks were identified by comparison of their retention times with appropriate FA methyl esters standards (Sigma–Aldrich), and FA concentrations were determined in relation to the peak area of internal standard.

Foetal adiposity was estimated by dividing the whole foetus total FA content by the foetal weight.

### Protein extracts for Western blotting

Placental tissue of 30 mg was homogenized in 0.3 mL ice-cold lysis buffer (20 mM Tris-HCl pH 7.5, 150 mM NaCl, 5 mM Na_2_EDTA, 1 mM EGTA, 1% Triton, 2.5 mM sodium pyrophosphate, 1 mM beta-glycerophosphate, 1 mM Na_3_VO_4_, 1 μg/mL leupeptin) from Cell Signaling Technology. Phenylmethanesulfonyl fluoride solution of 1 mM was added to the lysis buffer before homogenization (Ruiz-Alcaraz* et al.* 2005). Samples were homogenized using a Tissue Lyser LT device (Qiagen Iberia SL). Protein lysates were obtained from the supernatant after 15 min centrifugation at 10,000 ***g*** 4°C. Protein was quantified by Bradford assay (Bradford 1976), and samples were stored at −80°C until Western blot analysis.

### Western blot analysis

The primary antibodies used were rabbit polyclonal antibody against the orphan transporter called MFSD2a (Abcam), rabbit monoclonal against FA translocase (FAT) (Abcam), rabbit monoclonal against FATP-4 (Abcam), rabbit polyclonal antibody against adipocyte FA binding protein (A-FABP) (Sigma–Aldrich), rabbit monoclonal against phosphorylated AKT (Abcam), rabbit polyclonal against extracellular signal-regulated kinase (ERK) (Proteintech, Manchester, UK) and mouse monoclonal anti-beta-actin (Sigma–Aldrich). Anti-mouse and anti-rabbit secondary antibodies conjugated with horseradish peroxidase were obtained from Santa Cruz Biotechnology. Protein extracts (15 μg protein) diluted in sample buffer were resolved on 10% polyacrylamide gels and transferred onto PVDF membranes (Merck Millipore). Membranes were then blocked in phosphate saline buffer with 0.05% Tween 20 (PBS-T) containing 2% BSA for 1 h at room temperature. Thereafter, membranes were incubated with primary antibodies overnight at 4°C. Blots were then washed with PBS-T and probed for 1 h at room temperature with the correspondent secondary antibodies conjugated with horseradish peroxidase. Finally, membranes were stripped with Tris/HCl buffer pH 2.3 containing beta-mercaptoethanol 0.1 M and re-probed with anti-beta-actin to perform loading controls. Proteins were detected using a chemioluminescence kit according to the manufacture’s instruction (Pierce ECL 2 Western Blotting Substrate; Thermo Fisher Scientific) (Prieto-Sanchez* et al.* 2017). Density of all bands was determined by densitometry using Image Quant LAS 500 software (GE Healthcare). Relative protein expression data were normalized for beta-actin expression.

### Levels of inflammatory cytokines interleukin-1*β* (IL-1*β*), interleukin-6 (IL-6), tumor necrosis factor-a (TNF-a), and nitrated proteins

Renal cortex was homogenized in ice-cold 50 mmol/L Tris–HCl buffer pH 7.4 containing 1% NP-40, 0.25% sodium deoxycholate, 1 mmol/L EDTA, and 10% protease inhibitor cocktail (Sigma–Aldrich). Kidney homogenates were then centrifuged (10,000 *g* for 30 min) and stored at −80°C until analysis.

Concentrations of IL-1β, IL-6, TNF-*α* in renal cortex homogenates from each group were determined using a commercially available custom 32-plex Procartaplex Multiplex Immunoassay (Thermo Fisher Scientific) according to the manufacturer’s instructions with some modifications. Twenty-five microlitre of the kit-provided 1× Universal Assay Buffer was added to each well followed by 25 μL of four-fold serial diluted standards or undiluted samples into designated wells. After the addition of detection antibody, streptavidin-PE, and resuspend the beads, the test plate was prepared for analysis on a Luminex instrument by adding 120 μL of reading buffer. Finally, the plate was analysed on the Luminex MAGPIX System (Software: xPonent 4.2; Merck KGaA). The results were expressed as picogram/milligram of protein.

Extracted proteins from renal cortex homogenates (700 µg) were assayed for the quantitative measurement of nitrated proteins as an index of oxidative stress by using the Nitrotyrosine ELISA test kit (Hycult Biotechnology, Uden, The Netherlands) as previously published (Rodriguez* et al.* 2011).

### Total antioxidant activity

Total antioxidant activity (TAA) was determined in plasma and kidney of adult offspring rats according to Arnao *et al.* method (Arnao* et al.* 1999). The method is based on the ability of the antioxidants in the sample to reduce the radical cation of 2,2′-azino-bis-(3-ethylbenzthiazoline-6-sulphonic acid) (ABTS), determined by the decolouration of the ABTS cation radical (ABTS·+). ABTS·+ was generated in media containing 240 µL phosphate buffer (50 mM pH 7.5), ABTS 2 mM, H_2_O_2_ 60 µM, and horseradish peroxidase 0.25 µM. At stable ABTS·+ solutions, 10 µL of plasma or protein homogenate (kidney) were added, measuring the quenching of the absorbance at 730 nm after 6 min of reaction. Antioxidant activity was calculated by comparing the values of the sample with a standard curve of Trolox and expressed as Trolox millimoles equivalents by grams of albumin/protein (eq. Trolox millimoles/grams albumin or protein).

### Eicosanoids analysis in urine samples

*n*-6 and n-3 LC-PUFA metabolites 8-iso-prostaglandin F_2_*_α_* (8-iso-PGF_2_*_α_*), PGD_2_, PGE_2_, PGF_2_, PGD_3_, PGF_3_, Lipoxin A_4_ (LxA_4_), 15R-LxA_4_, Lx-B_4_, 5-hydroxy-6E,8Z,11Z,14Z-eicosatetraenoic acid (5-HETE), 12-hydroxyicosa-5Z,8Z,10E,14Z-tetraenoic acid (12-HETE), 15-hydroxyicosa-5Z,8Z,11Z,13E-tetraenoic acid (15-HETE), and the deuterated internal standard PGE_2_-d_4_ were purchased from Cayman Chemical Co. (Ann Arbor, MI, USA). All solvents and reagents used were HPLC grade.

Solid-phase extraction procedure of eicosanoids using Strata™ X-AW cartridges 200 mg/3mL (Phenomenex, Torrance, CA, USA) was performed in urine samples as previously described by Taylor *et al.* (2008). In brief, urine samples were centrifuged at 1400 ***g*** for 5 min at room temperature. The supernatant (4 mL) was mixed with ultra-pure water (4 mL) and internal standard (PGE_2_-d_4_ 50 ng in methanol). The sample pH was adjusted to 6.0 ± 0.05. Cartridges were preconditioned with 4 mL of methanol containing 2% formic acid (v/v) and subsequently equilibrated with 4 mL water. The diluted urine sample (8 mL) was then loaded onto the cartridge. The cartridge was washed with 4 mL water, followed by 8 mL methanol 25% (in water, v/v) and 4 mL methanol 50% (in water, v/v). Elution was subsequently performed with 3 mL of methanol containing 2% formic acid (v/v), 3 mL of methanol, and followed by 2 mL of methanol containing 50% acetonitrile (v/v). Cartridges were then dried under nitrogen gas and reconstituted in 50 μL methanol containing 0.1% formic acid (v/v) and injected onto the UHPLC-MS.

Chromatographic separation was carried out using a UPLC 1290 Infinity Series system (Agilent Technologies) equipped with a triple quadrupole mass spectrometer (6460 Jet Stream Series, Agilent Technologies) based on the methodology described by Le Faouder *et al.* (2013). An Excel C18 column (100 mm × 3.0 mm i.d., 1.7 µm) (ACE, Aberdeen, UK) was used. Limits of quantification and detection were also previously reported (Le Faouder* et al.* 2013). The relative standard deviation for peak area was in the range of 0.5–4.7% in the intraday test and 1.3–3.5% in the case of the inter-day test.

Standard curves were constructed at five concentrations for all compounds (0.025–0.5 µg/mL). The metabolite concentrations in urine were corrected for creatinine, which was measured using a commercial kit (CREATININE-J, Spinreact, Girona, Spain).

### Statistical analysis

Sample size was estimated based on HbAlc percentages in diabetic mice treated with AdipoRon published by Choi *et al.* (2018). Type I error was set at *α* = 0.05 and type II error *β* = 0.2 (power 80%), obtaining a minimum sample size of 11 animals per group. The software used for this estimation was nQuery 7.0 (Statsols HQ, Cork, Ireland).

The three experimental groups were compared using one-way ANOVA followed by a *post hoc* Bonferroni test. *P* < 0.05 was considered statistically significant. The results are expressed as the mean ± s.e.m. Statistical analysis was performed using SPSS, version 24.0 (IBM Corp.).

## Results

**Figure 1:**
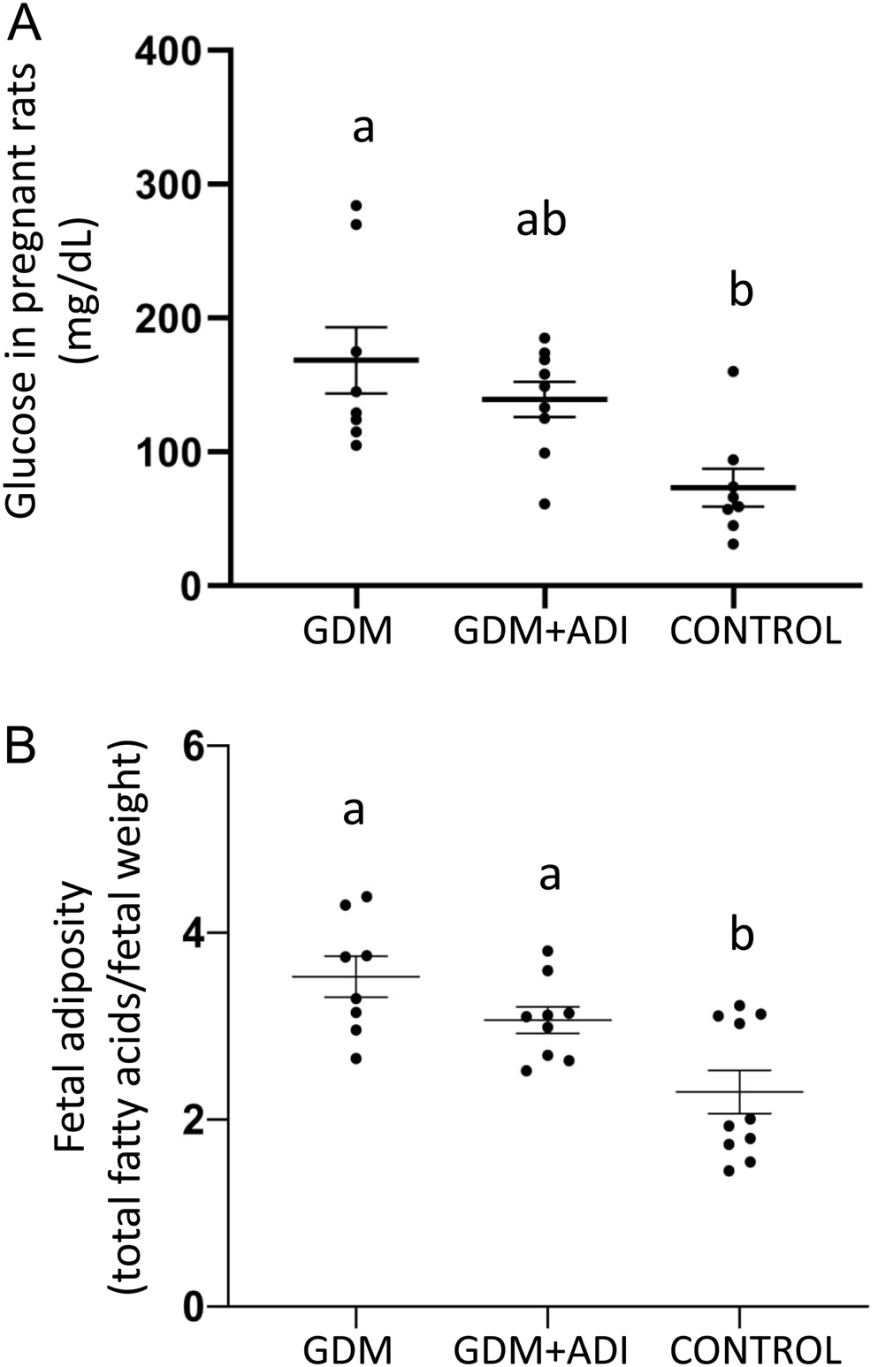
(A) Fasting glucose values at day 20 of gestation in rats with gestational diabetes (GDM), GDM rats treated with adiponectin agonist (AdipoRon) (GDM + ADI), and healthy control animals. (B) Total fat content/weight ratio of foetus at delivery. GDM *n* = 8, GDM + ADI *n* = 9, control *n* = 10. Results are expressed as means ± s.e.m. Different letters indicate significant differences between experimental groups (*P* < 0.05).

Pregnant diabetic rats (GDM) had significantly higher glycaemic levels than controls for both glucose (Fig. 1A) and HbAlc (Table 1). Adiponectin agonist (AdipoRon) administration tended to reduce glycaemia in diabetes-treated mothers compared to non-treated dams (Fig. 1A), following HbAlc the same non-significant trend (*P* = 0.073 GDM vs GDM + ADI). Maternal serum lipids (total cholesterol, HDL and LDL cholesterol, and TG) were similar in the three groups of animals (Table 1). Foetus from diabetic dams (GDM and GDM + ADI) presented intrauterine growth restriction compared to controls (Table 1). However, foetal fat content/foetal weight ratio indicated higher foetal adiposity in offspring of diabetic mothers, and AdipoRon tried to counteract this foetal fat accumulation (Fig. 1B).

**Table 1:** Maternal, foetal, and adult offspring parameters.

	GDM (*n* = 8)	GDM+ADI (*n* = 9)	Control (*n* = 10)	*P*
Maternal parameters (day 20 of gestation)				
Weight (g)	298.50 ± 7.74	316.40 ± 7.18	317.90 ± 12.82	0.350
HbA1c (%)	5.03 ± 0.12^a^	4.68 ± 0.13^a^	4.21 ± 0.11^b^	**0.001**
Cholesterol (mg/dL)	90.50 ± 7.47	101.78 ± 6.51	94.89 ± 6.83	0.481
HDL cholesterol (mg/dL)	31.50 ± 5.98	29.67 ± 6.10	19.56 ± 3.61	0.209
LDL cholesterol (mg/dL)	61.63 ± 11.02	67.78 ± 10.57	75.33 ± 8.33	0.598
TG (mg/dL)	448.88 ± 81.84	458.00 ± 61.63	493.22 ± 78.33	0.893
Albumin (g/dL)	2.45 ± 0.18	2.62 ± 0.10	2.38 ± 0.23	0.570
TAA (eq. Trolox mM/g albumin)	1.04 ± 0.06	0.89 ± 0.10	1.22 ± 0.34	0.567
Placental weight (g)	0.45 ± 0.01^a^	0.51 ± 0.02^b^	0.49 ± 0.02^ab^	**0.010**
Foetal parameters	(*n* = 8)	(*n* = 9)	(*n* = 10)	
Weight (g)	1.95 ± 0.06^a^	2.10 ± 0.05^a^	2.84 ± 0.27^b^	**0.002**
Foetus number	14.75 ± 0.59	13.60 ± 0.78	14.20 ± 0.84	0.590
Total fat (mg/g)	6.83 ± 0.33	6.43 ± 0.24	6.02 ± 0.21	0.102
Adult offspring (3 months old)	(*n* = 16)	(*n* = 14)	(*n* = 11)	
Weight (g)	300.44 ± 16.35	304.29 ± 16.87	293.55 ± 16.95	0.600
Glucose (mg/dL)	77.45 ± 3.25	80.23 ± 4.01	75.21 ± 1.96	0.777
Cholesterol (mg/dL)	82.55 ± 1.67	80.50 ± 4.02	69.13 ± 2.37	0.291
HDL Cholesterol (mg/dL)	57.18 ± 2.10	58.93 ± 3.58	48.31 ± 2.3	0.685
LDL Cholesterol (mg/dL)	12.64 ± 1.24^a^	9.50 ± 1.01^ab^	8.00 ± 0.92^b^	**0.026**
TG (mg/dL)	62.45 ± 5.11	60.07 ± 2.55	64.44 ± 4.72	0.846
Albumin (g/dL)	3.75 ± 0.11^a^	2.88 ± 0.24^b^	3.80 ± 0.06^a^	**0.002**

Results are expressed as means ± s.e.m.

Different superscript letters indicate significant differences between experimental groups (*P* < 0.05) (bold face).

GDM, gestational diabetes mellitus; GDM + ADI, animals with gestational diabetes mellitus treated with AdipoRon; TAA, total antioxidant activity.

No major differences were observed in maternal FA plasma, placenta, or foetal tissues (Table 2). In maternal plasma, eicosapentaenoic acid (20:5 n-3, EPA) was reduced in GDM mothers compared to control while DHA did not change significantly (Table 2). In placenta, DHA tended to increase by GDM (*P* = 0.107) while EPA again decreased significantly. However, no differences were observed in foetal brain (Table 2). Placental MFSD2a expression did not show differences between groups (GDM: 0.78 ± 0.12; GDM + ADI: 0.73 ± 0.10; control: 0.89 ± 0.15 arbitrary units, *P* = 0.636). Likewise, other FA transport proteins (FAT, FATP-4, and A-FABP) or proteins involved in insulin signalling (p-AKT and ERK) analysed in the placenta showed similar results between groups (Supplementary Fig. 1, see section on supplementary materials given at the end of this article).

**Table 2:** Maternal plasma, placenta, total foetus, and foetal brain fatty acid profile at day 20 of gestation.

Fatty acids (g/100 g fatty acids)	GDM (*n* = 8)	GDM+ADI (*n* = 9)	Control (*n* = 10)	*P*
Maternal plasma				
16:0	21.07 ± 0.91	20.29 ± 0.40	20.87 ± 0.52	0.605
18:0	8.73 ± 0.77	9.24 ± 0.61	8.98 ± 0.36	0.807
18:1 n-9	14.26 ± 1.21	15.35 ± 0.75	14.07 ± 0.78	0.498
18:2 n-6	21.32 ± 1.19	20.26 ± 0.76	20.55 ± 1.02	0.723
20:4 n-6	15.04 ± 0.85	16.59 ± 0.81	17.49 ± 1.04	0.173
20:5 n-3 (EPA)	0.15 ± 0.05^a^	0.27 ± 0.06^ab^	0.39 ± 0.05^b^	**0.016**
22:6 n-3 (DHA)	6.70 ± 0.71	5.84 ± 0.35	5.60 ± 0.46	0.268
SFA	31.72 ± 1.63	31.50 ± 0.58	31.97 ± 0.53	0.923
MUFA	18.55 ± 1.44	20.13 ± 0.81	18.55 ± 0.95	0.417
PUFA	49.65 ± 1.49	48.21 ± 0.81	49.34 ± 1.01	0.576
n-6/*n*-3	5.41 ± 0.51	6.00 ± 0.30	6.30 ± 0.48	0.336
LC-PUFA n-6	19.91 ± 0.97	20.34 ± 0.73	21.13 ± 1.04	0.615
LC-PUFA n-3	7.81 ± 0.80	6.88 ± 0.38	6.78 ± 0.53	0.355
Placenta				
16:0	21.49 ± 0.28^ab^	20.98 ± 0.31^a^	21.98 ± 0.18^b^	**0.020**
18:0	16.87 ± 0.23	16.87 ± 0.37	16.01 ± 0.33	0.087
18:1 n-9	9.85 ± 0.54	9.43 ± 0.31	9.34 ± 0.14	0.512
18:2 n-6	15.27 ± 0.52	14.79 ± 0.46	13.82 ± 0.46	0.086
20:4 n-6	16.50 ± 0.56^a^	17.99 ± 0.35^b^	17.85 ± 0.35^ab^	**0.028**
20:5 n-3 (EPA)	0.08 ± 0.01^a^	0.10 ± 0.01^ab^	0.13 ± 0.01^b^	**0.006**
22:6 n-3 (DHA)	4.55 ± 0.20	4.59 ± 0.10	4.23 ± 0.12	0.107
SFA	41.63 ± 0.19	41.21 ± 0.39	41.73 ± 0.44	0.538
MUFA	14.11 ± 0.63	13.70 ± 0.41	13.74 ± 0.21	0.735
PUFA	44.14 ± 0.62	44.96 ± 0.37	44.37 ± 0.35	0.390
n-6/*n*-3	7.21 ± 0.26	7.30 ± 0.18	7.71 ± 0.17	0.142
LC-PUFA n-6	23.16 ± 0.94	24.44 ± 0.49	25.17 ± 0.29	0.053
LC-PUFA n-3	5.20 ± 0.21	5.24 ± 0.11	4.93 ± 0.12	0.236
Total foetus				
16:0	23.73 ± 0.43	23.13 ± 0.26	23.82 ± 0.59	0.491
18:0	12.10 ± 0.18	12.28 ± 0.16	12.63 ± 0.16	0.072
18:1 n-9	14.82 ± 0.21	14.76 ± 0.22	14.55 ± 0.23	0.631
18:2 n-6	8.91 ± 0.49	8.20 ± 0.46	7.80 ± 0.25	0.139
20:4 n-6	14.83 ± 0.22	15.59 ± 0.28	15.11 ± 0.35	0.194
20:5 n-3 (EPA)	0.11 ± 0.01^ab^	0.11 ± 0.01^a^	0.13 ± 0.01^b^	**0.020**
22:6 n-3 (DHA)	6.12 ± 0.13	6.32 ± 0.13	6.02 ± 0.17	0.318
SFA	38.25 ± 0.47	38.16 ± 0.30	39.51 ± 0.58	0.069
MUFA	24.00 ± 0.30	23.93 ± 0.45	23.77 ± 0.37	0.892
PUFA	37.53 ± 0.40	37.69 ± 0.50	36.49 ± 0.68	0.225
n-6/*n*-3	4.44 ± 0.12	4.34 ± 0.08	4.37 ± 0.06	0.712
LC-PUFA n-6	21.30 ± 0.30	22.06 ± 0.28	21.51 ± 0.38	0.237
LC-PUFA n-3	6.64 ± 0.13	6.78 ± 0.13	6.52 ± 0.19	0.467
Foetal brain				
16:0	29.97 ± 0.51	31.16 ± 0.53	31.65 ± 0.69	0.124
18:0	15.08 ± 0.37	14.62 ± 0.13	14.80 ± 0.37	0.578
18:1 n-9	12.45 ± 0.44	11.92 ± 0.16	11.99 ± 0.18	0.319
18:2 n-6	1.33 ± 0.16^a^	1.03 ± 0.07^ab^	0.94 ± 0.09^b^	**0.036**
20:4 n-6	11.85 ± 1.82	13.64 ± 0.20	12.96 ± 0.31	0.141
20:5 n-3 (EPA)	0.09 ± 0.10	0.03 ± 0.03	0.03 ± 0.03	0.974
22:6 n-3 (DHA)	9.87 ± 0.35	10.23 ± 0.24	10.00 ± 0.40	0.738
SFA	45.77 ± 0.23	46.38 ± 0.47	47.20 ± 0.71	0.210
MUFA	22.89 ± 1.94	20.34 ± 0.27	20.53 ± 0.34	0.398
PUFA	31.11 ± 1.95	33.04 ± 0.48	32.01 ± 0.85	0.477
n-6/*n*-3	2.08 ± 0.24	2.17 ± 0.06	2.14 ± 0.05	0.477
LC-PUFA n-6	19.34 ± 2.22	21.42 ± 0.30	20.67 ± 0.50	0.412
LC-PUFA n-3	10.06 ± 0.42	10.32 ± 0.27	10.07 ± 0.41	0.786

Results are expressed as means ± s.e.m. Only selected fatty acids are listed but all fatty acids analysed were considered for the calculation of percentages.

Different superscript letters indicate significant differences between experimental groups (*P* < 0.05) (bold face).

DHA, docosahexaenoic acid; EPA, eicosapentaenoic acid; GDM, gestational diabetes mellitus; GDM + ADI, animals with gestational diabetes mellitus treated with AdipoRon; LC-PUFA, long-chain polyunsaturated fatty acids (>18 carbons); MUFA, monounsaturated fatty acids; PUFA, polyunsaturated fatty acids; SFA, saturated fatty acids.

**Figure 2:**
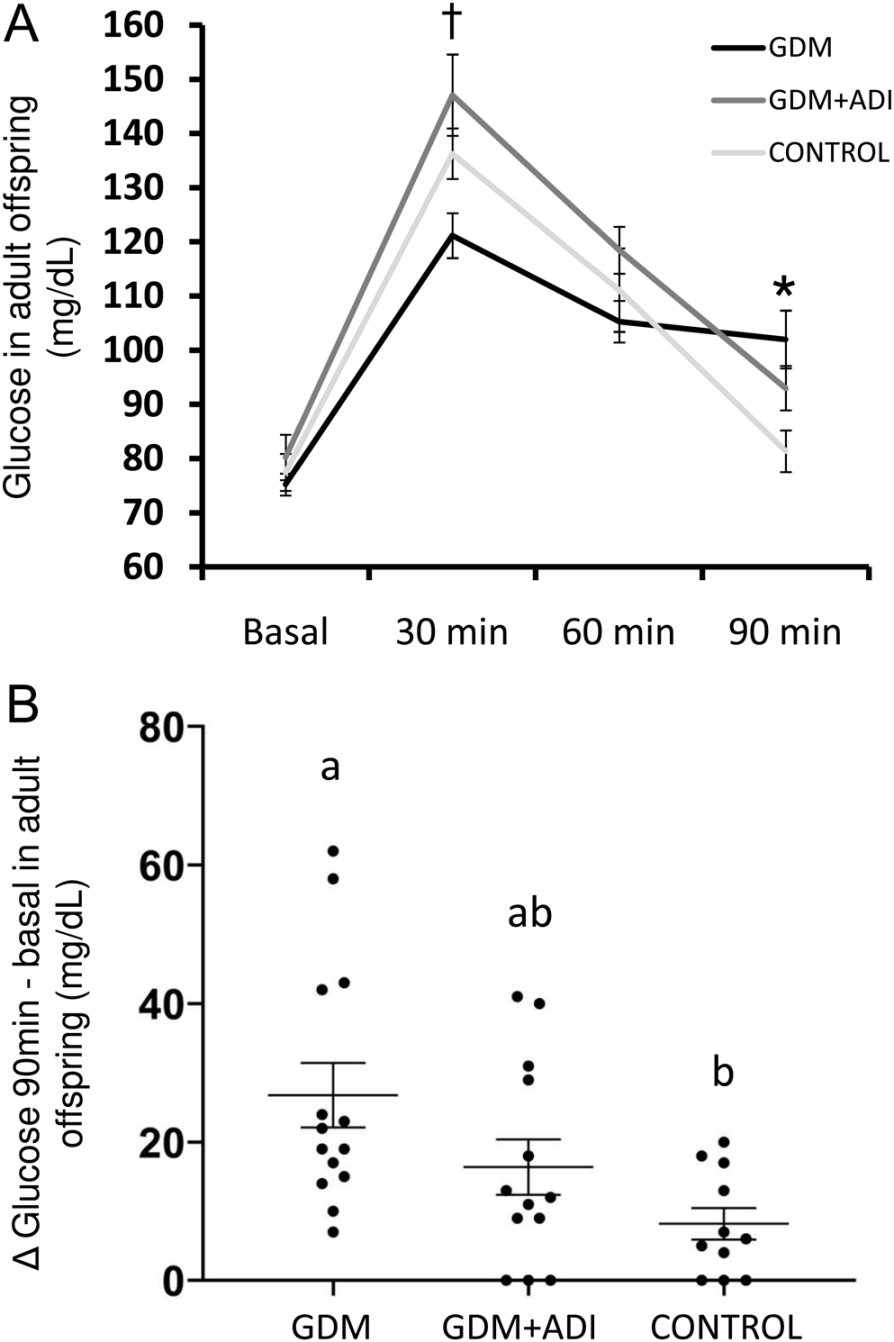
(A) Glucose values after an oral glucose overload (2 g/kg) in the adult offspring of rats with gestational diabetes (GDM), GDM rats treated with adiponectin agonist (AdipoRon) (GDM + ADI), and healthy control animals. (B) Glucose increase between basal and 90 min postchallenge (2 g glucose/kg) in the adult offspring. GDM *n* = 16, GDM+ADI *n* = 14, control *n* = 11. Results are expressed as means ± s.e.m. *Indicates significant differences between GDM and control groups (*P* < 0.05). ^†^Indicates significant differences between GDM and GDM+ADI groups (*P* < 0.05). Different letters indicate significant differences between experimental groups (*P* < 0.05).

At the adult state, offspring of diabetic and control mothers showed similar fasting glucose levels (Table 1). GDM offspring showed lower glucose concentration than GDM + ADI ones after 30 min of an oral glucose overload test (Fig. 2A). However, GDM offspring had significantly higher glucose increase between basal and 90 min post-challenge compared to controls (*P* = 0.009) while a positive effect of AdipoRon was observed on reducing this basal- 90 min glucose increase in the offspring (Fig. 2B). Both GDM + ADI and control were returning to basal values of glycaemia after 90 min while GDM still presented elevated values of serum glucose (Fig. 2A). Serum LDL cholesterol was also higher in the offspring of GDM mothers compared to the control group while no differences were found in LDL cholesterol or triglycerides level (Table 1). GDM + ADI showed lower serum albumin than GDM or control in adult offspring (Table 1).

**Figure 3:**
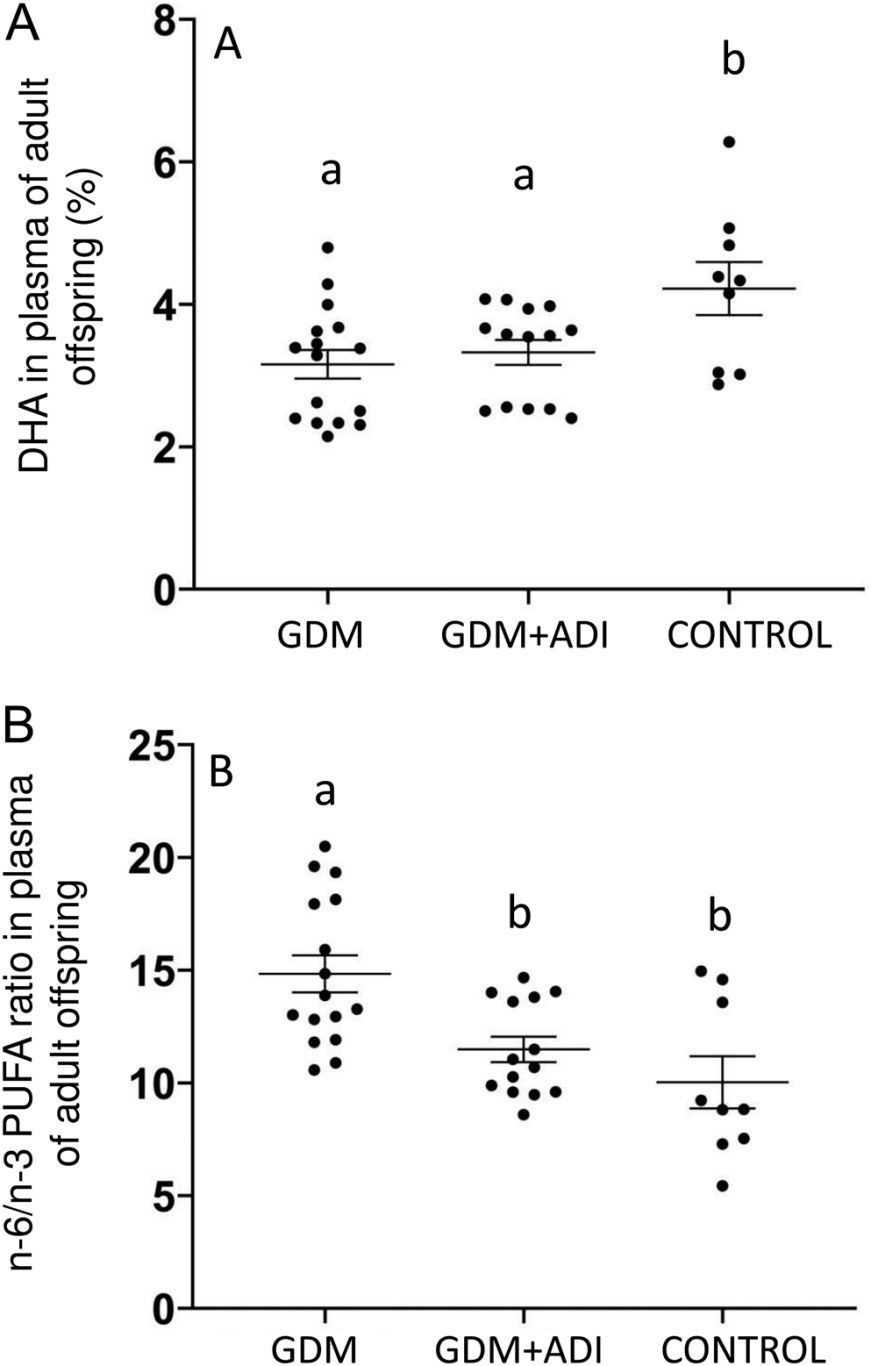
(A) Docosahexaenoic acid (DHA) in plasma of adult offspring (3 months old) of rats with gestational diabetes (GDM), GDM rats treated with adiponectin agonist (AdipoRon) (GDM + ADI), and healthy control animals. (B) n-6/*n*-3 polyunsaturated fatty acids (PUFA) ratio in plasma of adult offspring. GDM *n* = 16, GDM + ADI *n* = 14, Control *n* = 11. Results are expressed as means ± s.e.m. Different letters indicate significant differences between experimental groups (*P* < 0.05).

Concerning to FA profile in the adult offspring, DHA was significantly reduced in plasma of GDM offspring compared to control (Fig. 3A), whereas arachidonic acid (20:4 n-6) (Table 3) and n-6/*n*-3 LC-PUFA ratio increased (Fig. 3B). AdipoRon normalized n-6/*n*-3 LC-PUFA ratio in the GDM offspring to values observed in control animals (Fig. 3B). Adult brain FA composition remained similar among groups, showing the strong regulation of its FA composition (Table 3).

**Table 3:** Fatty acid profile in plasma and brain of 3 months old offspring.

Fatty acids (g/100 g fatty acids)	GDM (*n* = 16)	GDM+ADI (*n* = 14)	Control (*n* = 11)	*P*
16:0	22.74 ± 0.56	22.58 ± 0.65	20.75 ± 0.77	0.383
18:0	13.77 ± 0.63	15.54 ± 0.78	15.82 ± 0.79	0.528
18:1 n-9	9.16 ± 0.43^a^	5.95 ± 0.33^b^	10.63 ± 0.53^c^	**<0.001**
18:2 n-6	15.36 ± 0.77	12.02 ± 0.71	11.80 ± 1.01	0.184
20:4 n-6	29.87 ± 1.02^a^	29.81 ± 0.70^ab^	27.47 ± 1.33^b^	**0.045**
SFA	38.60 ± 0.52^a^	43.04 ± 0.82^b^	39.93 ± 0.66^ab^	**0.007**
MUFA	12.47 ± 0.66^a^	9.38 ± 0.47^a^	15.45 ± 0.86^b^	**<0.001**
PUFA	48.92 ± 0.60^a^	47.53 ± 0.62^a^	44.61 ± 1.45^b^	**0.001**
LC-PUFA n-6	30.00 ± 0.98^ab^	30.97 ± 0.71^a^	28.09 ± 1.25^b^	**0.038**
LC-PUFA n-3	3.20 ± 0.19	3.67 ± 0.18	4.27 ± 0.39	**0.110**
Brain				
16:0	17.69 ± 0.14	17.91 ± 0.11	17.79 ± 0.12	0.464
18:0	19.38 ± 0.08	19.87 ± 0.17	19.66 ± 0.10	0.150
18:1 n-9	16.04 ± 0.17	15.79 ± 0.12	16.34 ± 0.10	0.119
18:2 n-6	0.72 ± 0.02	0.70 ± 0.02	0.72 ± 0.02	0.819
20:4 n-6	10.63 ± 0.11	10.56 ± 0.07	10.56 ± 0.11	0.973
22:6 n-3 (DHA)	17.18 ± 0.17	17.36 ± 0.15	17.15 ± 0.16	0.694
SFA	40.27 ± 0.13^b^	41.17 ± 0.23^a^	40.70 ± 0.12^ab^	**0.037**
MUFA	25.21 ± 0.31	24.22 ± 0.24	24.79 ± 0.31	0.120
PUFA	34.42 ± 0.22	34.55 ± 0.17	34.36 ± 0.20	0.596
n-6/*n*-3	0.90 ± 0.01	0.87 ± 0.01	0.90 ± 0.01	0.337
LC-PUFA n-6	15.50 ± 0.14	15.38 ± 0.11	15.50 ± 0.14	0.820
LC-PUFA n-3	17.47 ± 0.16	17.67 ± 0.15	17.44 ± 0.16	0.623

Results are expressed as means ± s.e.m. 3 months old offspring from 3 to 4 pregnant rats per group. Only selected fatty acids are listed but all fatty acids analysed were considered for the calculation of percentages. GDM *n* = 16 (8 males/8 females), GDM + ADI *n* = 14 (6 males/8 females), Control *n* = 11 (6 males/5 females).

Different superscript letters indicate significant differences between experimental groups (*P* < 0.05) (bold face).

DHA, docosahexaenoic acid; GDM, gestational diabetes mellitus; GDM + ADI, animals with gestational diabetes mellitus treated with AdipoRon; LC-PUFA, long-chain polyunsaturated fatty acids (>18 carbons); MUFA, monounsaturated fatty acids; PUFA, polyunsaturated fatty acids; SFA, saturated fatty acids.

Maternal AdipoRon treatment tended to increase total antioxidant status, determined by TAA, in the kidney of GDM + ADI offspring (*P* = 0.086) (Table 4). This was confirmed with the lower nitrotyrosine level (oxidative stress marker) observed in the renal cortex of these animals (Table 4). Moreover, AdipoRon significantly reduced pro-inflammatory cytokines IL-1*β*, IL-6, and TNF-*α* in renal tissue of adult offspring with respect to both GDM and control groups (Table 4). However, PG and LC-PUFA-derived eicosanoids did not show major differences in the urine of adult offspring (Table 4).

**Table 4:** Oxidative stress parameters, immunological markers, prostaglandins, and LC-PUFA intermediate metabolites in adult offspring.

	GDM (*n* = 16)	GDM+ADI (*n* = 14)	Control (*n* = 11)	*P*
Plasma				
TAA (eq. Trolox mM/g albumin)	1.02 ± 0.03	1.50 ± 0.27	1.04 ± 0.03	0.307
Renal cortex				
TAA (eq. Trolox mM/mg protein)	54.01 ± 1.43	62.84 ± 4.46	49.13 ± 1.18	0.086
IL-1*β* (pg/mg protein)	276.16 ± 23.72^a^	211.77 ± 18.10^b^	270.23 ± 16.78^ab^	**0.039**
IL-6 (pg/mg protein)	202.51 ± 18.98^a^	132.57 ± 10.85^b^	212.44 ± 16.39^a^	**0.046**
TNF-*α* (pg/mg protein)	152.31 ± 13.99^a^	111.45 ± 10.69^b^	151.36 ± 11.52^a^	**0.047**
Nitrotyrosine (pmol/mg protein)	6.69 ± 0.33^a^	5.47 ± 0.23^b^	6.60 ± 0.27^a^	**0.024**
Total protein (mg/mL)	24.17 ± 0.87^a^	33.71 ± 1.78^b^	25.86 ± 0.93^a^	**0.001**
Urine (ng/mg creatinine)				
8-Iso-15(R)-PGF_2__α_	0.03 ± 0.01	0.03 ± 0.01	0.04 ± 0.01	0.939
PGD_2_	3.98 ± 0.98	3.91 ± 1.10	4.96 ± 1.30	0.542
PGE_2_	2.75 ± 0.53	2.01 ± 0.33	1.89 ± 0.33	0.654
PGF_2_	2.72 ± 0.55	2.08 ± 0.55	1.86 ± 0.44	0.400
PGD_3_	0.11 ± 0.04	0.03 ± 0.01	0.05 ± 0.03	0.379
PGF_3_	0.43 ± 0.14	0.13 ± 0.06	0.36 ± 0.13	0.146
LxA_4_	0.36 ± 0.09	0.37 ± 0.13	0.38 ± 0.10	0.985
15R-LxA_4_	0.43 ± 0.11	0.48 ± 0.16	0.35 ± 0.10	0.873
Lx-B_4_	0.08 ± 0.02	0.05 ± 0.02	0.09 ± 0.03	0.702
5-HETE	0.35 ± 0.16	0.25 ± 0.03	0.17 ± 0.03	0.771
12-HETE	0.54 ± 0.12	0.34 ± 0.07	0.27 ± 0.05	0.257
15-HETE	1.05 ± 0.27	0.46 ± 0.09	0.81 ± 0.19	0.517

Results are expressed as means ± s.e.m. 3 months old offspring from 3 to 4 pregnant rats per group. GDM *n* = 16 (8 males/8 females), GDM + ADI *n* = 14 (6 males/8 females), control *n* = 11 (6 males/5 females).

Different superscript letters indicate significant differences between experimental groups (*P* < 0.05) (bold face).

HETE, hydroxyicosa-tetraenoic acid; IL, interleukin; Lx, lipoxin; PG, prostaglandin; TAA, total antioxidant activity; TNF-*α*, tumor necrosis factor *α*.

## Discussion

Here we report the effects of the orally active adiponectin agonist termed AdipoRon on glycaemia and lipid profile in both mother and adult offspring, after its administration during gestation to diabetic rats. As expected, diabetic animals presented higher glucose and HbAlc than non-diabetic control animals. However, AdipoRon treatment tended to reduce glycaemia, indicating a positive effect of the adiponectin agonist on glucose control in the mothers and offspring. It is worth to mention that diabetic animals received daily exogenous insulin which might have affected the results observed. Okada-Iwabu *et al.* reported a significant reduction in fasting plasma glucose and insulin levels of high-fat diet-induced obese mice after the administration of AdipoRon for 10 days (Okada-Iwabu* et al.* 2013). They found increased exercise endurance and increased expression of genes involved in FA oxidation in muscle (e.g. medium-chain acyl-CoA dehydrogenase) and lower activation of gluconeogenesis pathways in the liver (e.g. glucose-6-phosphatase) of animals treated with AdipoRon (Okada-Iwabu* et al.* 2013). Low levels of serum adiponectin are negatively and independently associated with metabolic syndrome-related disorders such as insulin resistance, diabetes, and dyslipidaemia (Kishida* et al.* 2012). Thus, administration of AdipoRon during gestation may be a useful strategy to improve glycaemic control in GDM subjects, reducing the risk of later glucose intolerance and insulin resistance in the offspring (Scholtens* et al.* 2019).

Diabetes can result in intrauterine growth restriction in animals. STZ-induced diabetes in rats can be associated with mild diabetes and macrosomia or with insulin-deficient diabetes and foetal growth restriction,the latter being the more common situation (Eriksson *et al.* 1984, Canavan & Goldspink 1988, Holemans *et al.* 1997, Ergaz *et al.* 2005). Despite intrauterine growth restriction, GDM foetus showed higher adiposity than controls, which tried to be mitigated by AdipoRon treatment. As no differences were detected in FA carriers expression in placenta, changes in foetal adiposity could be driven by maternal glucose transfer. There are some evidences of increased glucose transporters expression in diabetes-complicated pregnancies (Stanirowski* et al.* 2018). The independent-insulin glucose transporter 1 (GLUT-1) may be responsible for excessive glucose transfer and acceleration of foetal adiposity in the GDM foetus. Aye and colleagues reported reduced insulin receptors activity (IR-*β* and IRS-1), p-AKT, and mTORC1 activity in the placenta of obese pregnant mice after adiponectin administration while no differences in ERK expression (Aye* et al.* 2015). mTORC1 is a placental nutrient sensor activated by insulin that promotes nutrient transport across the placenta (especially amino acids) contributing to foetal growth (Jansson *et al.* 2012, Dimasuay *et al.* 2016). We were not able to quantify placental mTORC1 because of technical problems, but we did not find differences in p-AKT or ERK expression (Supplementary Fig. 1). Thus, AdipoRon may serve as a therapeutic treatment against negative foetal outcomes of GDM related to foetal adiposity. Growth-restricted foetus (GDM and GDM + ADI) showed higher levels of serum LDL cholesterol in adulthood (3 months old) while similar concentrations were observed for total and HDL cholesterol. Tenhola *et al.* (2000) reported negative associations between birthweight of small for gestational age infants and later total cholesterol concentration in blood during childhood, supporting the relevance of foetal nutrition to adult disease (Ornoy 2011).

Our group previously reported impaired placental transfer of ^13^C-DHA in GDM women compared to healthy women (Pagan* et al.* 2013). MFSD2a is postulated as one of the major transporters of Lyso-DHA across the blood–brain barrier (Nguyen* et al.* 2014) and also in the placenta (Prieto-Sanchez* et al.* 2017). In the present study, no differences were observed in MFSD2a expression in the placenta or DHA in the total foetus. However, DHA percentage was estimated in the whole foetus and not in cord blood, which could have mask differences in FA foetal plasma profile.

We demonstrated for the first time that GDM+ADI offspring presented an improved response to glucose overload than GDM offspring, indicating the effectiveness of the maternal treatment with AdipoRon on the long-term glucose tolerance in the next generation. However, it was surprising that GDM offspring showed better glucose tolerance than GDM + ADI offspring at 30 min post-challenge, which may be related to an alteration in gastric emptying (Goyal *et al.* 2019, Wu *et al.* 2020). Impaired insulin response and altered pancreatic *β*-cell function are thought to be responsible for the transgenerational transmission of GDM (Aerts & van Assche 2006). The use of AdipoRon in the treatment of GDM may lead to a lower incidence of metabolic syndrome and diabetes in future generations. Our results in pregnant rats agree with those recently published by Scholtens *et al.* (2019) which showed, across the maternal glucose spectrum, associations between the exposure to hyperglycaemia *in utero* and elevated glucose values and insulin resistance during childhood (HAPO follow-up cohort). In summary, both studies corroborate the long-term health consequences of maternal hyperglycaemia during pregnancy for the offspring. Reduced tolerance to glucose in adult offspring of GDM may contribute to the pathogenesis of metabolic syndrome and pre-diabetes or diabetes mellitus type 2 (Clausen* et al.* 2008).

Inflammatory cytokines and oxidative stress markers were measured in the renal cortex as kidneys’ function is usually impaired by diabetes. GDM + ADI offspring showed decreased pro-inflammatory cytokines (IL-1, IL-6, and TNF-*α*) in the renal cortex compared to GDM and control, which can be due in part to the higher protein content in the tissue. Our results are consistent with those reported by Okada-Iwabu *et al.* who showed a reduction in the expression levels of the genes encoding pro-inflammatory cytokines (e.g. IL-6 and TNF-*α*) in the liver and white adipose tissue of high-fat diet obese mice after AdipoRon administration (Okada-Iwabu* et al.* 2013). Oxidative stress status in kidneys of adult offspring was also reduced by AdipoRon treatment (higher TAA and lower nitrotyrosine). Nevertheless, no significant differences were observed in plasma TAA or urinary eicosanoids. Several studies have recently reported a reduction in renal oxidative stress, apoptosis, lipotoxicity, and inflammation after AdipoRon administration in animal models, which could be especially beneficial in the pathophysiology of diabetic nephropathy and other related disorders (Choi *et al.* 2018, Xiong *et al.* 2020, Qiu *et al.* 2021). This is probably mediated by activating the AMPK pathway (Kim *et al.* 2018, Gu *et al.* 2020). Thus, AdipoRon seems to promote positive effects on antioxidant and immune systems that could protect against the renal injury associated to diabetes and other pathologies in long term.

DHA was strongly reduced in plasma of GDM and GDM + ADI adult offspring. Reduced DHA status has been extensively described in the cord blood of GDM women (Min *et al.* 2005, Pagan *et al.* 2013, Ortega-Senovilla *et al.* 2020), but here we report the long-term deleterious consequences of GDM on the FA in the offspring. AdipoRon treatment during pregnancy did not improve the DHA level of GDM + ADI but was effective to reduce the unbalanced n-6/*n*-3 PUFA ratio observed in GDM offspring. In the present study, we cannot discard that this situation of lower DHA in GDM offspring was likely established already at delivery since cord blood samples were not available. Zhao *et al.* showed associations between low circulating DHA levels and compromised foetal insulin sensitivity, and this may be involved in the early programming of the susceptibility to type 2 diabetes in offspring of GDM women (Zhao* et al.* 2014). The AdipoRon administration during pregnancy may also have additional effects in the offspring under different metabolic challenges (e.g. high-fat diet) and this should be explored in future studies.

The strengths of the present study are the administration for the first time of the novel adiponectin agonist AdipoRon in pregnant diabetic animals and the evaluation of its effect for the offspring at both birth and adult state. Our study also presents some limitations since the animal model of diabetes induced by STZ used has some physiopathological differences with human GDM that may include insulin treatment requirement, absence of increased insulin resistance, or foetal growth restriction among others. We cannot exclude that these physiological differences in the rat model could have biased the results reported and the extrapolation to the human GDM condition.

## Conclusions

In conclusion, AdipoRon administration to pregnant diabetic rats led to improved maternal glycaemia and reduced foetal adiposity associated with GDM. At the adult state, GDM offspring presented impaired glucose response compared to control, and it was counteracted by AdipoRon treatment. In addition, AdipoRon improved the plasma n-6/*n*-3 ratio in the offspring and showed some positive effects on antioxidant and inflammatory status. Adiporon seems to be a promising therapeutic drug for the control of glycaemia in the diabetic mother and the offspring in long term.

## Supplementary Material

Supplemental Figure 1. Protein expression of the A) major facilitator superfamily domain-containing 2a (MFSD2a), B) fatty acid translocase (FAT), C) fatty acid transport protein 4 (FATP-4), D) adipocyte fatty acid binding protein (A-FABP) and E) phosphorylated AKT (p-AKT) and F) extracellular signal-regulated kinase (ERK) in placenta at day 20 of gestation in rats with gestational diabetes (GDM), GDM rats treated with adiponectin agonist (AdipoRon) (GDM+ADI) and healthy control animals. GDM n=8, GDM+ADI n=9, Control n=10. Results are expressed as means ± SEM. Different letters indicate significant differences between experimental groups (P <0.05).

## Declaration of interest

The authors declare that there is no conflict of interest that could be perceived as prejudicing the impartiality of the research reported.

## Funding

This study was financially supported by the Spanish Government (GD-BRAIN, SAF2015-69265-C2-1-R), the Maternal and Child Health and Development Research Network (RED SAMID III, RD 16/0022/0009) and the Research Excellence Group CHRONOHEALTH (Séneca Foundation, 19899/GERM/15, Murcia).

## Data availability

The datasets generated during and/or analysed during the current study are not publicly available but are available from the corresponding author on reasonable request.

## Author contribution statement

A G, F R, and E L designed and conducted the study, performed experiments, and wrote the manuscript. A G, F R, M S-C, L E M-G, and M D A-O performed laboratory analyses. P S-G and M B A acquired data. A G, F R, M S-C, M B A, and E L interpreted data. All authors have read and approved the final version of the manuscript and agreed to be accountable for all aspects of the work in ensuring that questions related to the accuracy or integrity of any part of the work are appropriately investigated and resolved.
