# Supplementary material for: Adiponectin agonist treatment in diabetic pregnant rats
Source: J Endocrinol. 2021 Jun 22;251(1):1–13. doi: 10.1530/JOE-20-0617 (PMC8345900; doi:10.1530/JOE-20-0617)
Supplement: Supplemental Figure 1. Protein expression of the A) major facilitator superfamily domain-containing 2a (MFSD2a), B) fatty acid translocase (FAT), C) fatty acid transport protein 4 (FATP-4), D) adipocyte fatty acid binding protein (A-FABP) and E) phosphorylated AKT (p-AKT) and F) extracellular signal [file supplementary_figure_1.pdf]

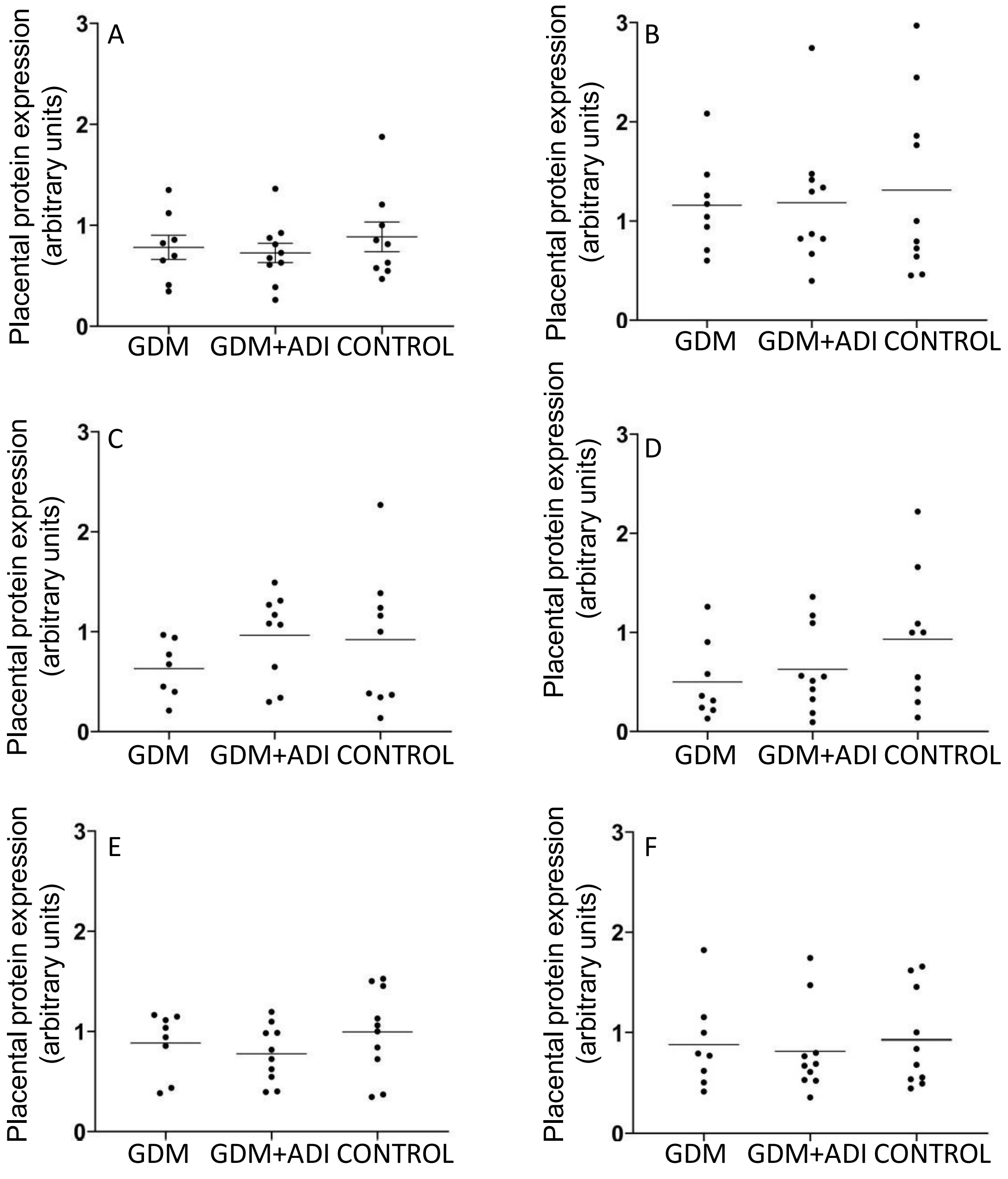

A
B
C
D
E
F
Placental protein expression (arbitrary units)
0
1
2
3
GDM
GDM+ADI
CONTROL
